# Supplementary material for: Cardiogenic Shock Integrated PHenotyping for Event Reduction: A Pilot Metabolomics Analysis
Source: Int J Mol Sci. 2023 Dec 18;24(24):17607. doi: 10.3390/ijms242417607 (PMC10743901; doi:10.3390/ijms242417607)
Supplement: Supplementary file 1 [file ijms-24-17607-s001.zip › CIPHER_pilot_metabolomics_analysis_Table_S1.doc]

**Supplementary Table 1. Comparison among T0, T1 and T2 in the 9 patients with all blood sample collection**

| **Variable** | **T0 (n=9)** | **T1 (n=9)** | **T2 (n=9)** | ***P*** |
| --- | --- | --- | --- | --- |
| IL-6 (pg/mL) | 52,43 ± 26,09 | 68,17 ± 43,42 | 35,24 ± 14,79 | 0.016 |
| Syndecan-1 (ng/mL) | 1,01 ± 0,97 | 1,59 ± 1,41 | 1,58 ± 2,16 | 0.197 |
| Heparan Sulfate (ng/mL) | 19,99 ± 30,30 | 24,36 ± 47,95 | 17,63 ± 23,04 | 0.717 |
| Angiopoietin-2 (pg/mL) | 274,61 ± 270,86 | 223,97 ± 135,44 | 354,54 ± 166,05 | 0.121 |

Friedman Test

IL-6, significantly decreased after hemodynamic stabilization.
